# Modeling Psychological Contract Violation using Dual Regime Models: An Event-based Approach

**DOI:** 10.3389/fpsyg.2017.01948

**Published:** 2017-11-07

**Authors:** Joeri Hofmans

**Affiliations:** Work and Organizational Psychology, Vrije Universiteit Brussel, Brussels, Belgium

**Keywords:** psychological contract, violation feelings, hurdle model, zero-inflated model, dual regime models

## Abstract

A good understanding of the dynamics of psychological contract violation requires theories, research methods and statistical models that explicitly recognize that violation feelings follow from an event that violates one's acceptance limits, after which interpretative processes are set into motion, determining the intensity of these violation feelings. Whereas theories—in the form of the dynamic model of the psychological contract—and research methods—in the form of daily diary research and experience sampling research—are available by now, the statistical tools to model such a two-stage process are still lacking. The aim of the present paper is to fill this gap in the literature by introducing two statistical models—the Zero-Inflated model and the Hurdle model—that closely mimic the theoretical process underlying the elicitation violation feelings via two model components: a binary distribution that models whether violation has occurred or not, and a count distribution that models how severe the negative impact is. Moreover, covariates can be included for both model components separately, which yields insight into their unique and shared antecedents. By doing this, the present paper offers a methodological-substantive synergy, showing how sophisticated methodology can be used to examine an important substantive issue.

Psychological contracts (PCs)—or the individual's perceptions of the mutual obligations of the employee and employer (Rousseau, 1995)—are critical for a good understanding of the employment relationship. That the PC takes a central place in the employee's working life has time and time again been demonstrated by research showing that PC breach—or the awareness that the employer fails to fulfill one or more obligations included in the PC—is associated with negative work-related attitudes and behaviors, such as reduced job satisfaction, lower commitment, decreased levels of performance, and increased turnover intentions (Zhao et al., 2007; Griep et al., 2016; Solinger et al., 2016).

These negative consequences can be explained by the fact that, when the employee notices that his/her organization fails to meet its obligations, (s)he is likely to develop an intense negative emotional reaction (i.e., violation feelings), which in turn has several negative attitudinal and behavioral consequences for both the employee and the organization (Morrison and Robinson, 1997; Vantilborgh et al., 2016). Thus, according to this reasoning, violation feelings result from a two-stage process in which the employee first engages in a cognitive evaluation of events taking place, after which negative emotions might follow from this cognitive evaluation.

Despite the general awareness that violation feelings result from a two-stage decision-making process in which the employee first assesses whether anything has violated his/her psychological contract and then evaluates the negative emotional impact of these potential violation(s), few studies have explicitly examined violation as a two-stage decision making process (for exceptions, see Griep et al., 2016; Bal et al., 2017). One important reason is that the large majority of empirical work on psychological contracts in general and violation feelings in particular has been cross-sectional in nature (Conway and Briner, 2002). These studies typically examine whether between-person differences in violation feelings relate to between-person differences in work outcomes by asking people to report about their violation experiences and work outcomes retrospectively, over an extended period of time. Whereas this has undoubtedly furthered our understanding of psychological contract violations, an important downside of these studies is that they fail to capture the temporal, dynamic nature of the employee-employer relationship. For example, because these studies do not focus on particular events but rather on general experiences over an extended period of time, they are unable to tap into the two-stage decision-making process of violation feelings.

This is an important limitation because a good understanding of the dynamics of psychological contract violation requires theories, research methods and statistical models that explicitly recognize that violation feelings follow from an event that violates one's acceptance limits, after which interpretative processes determine the intensity of these violation feelings. Whereas such dynamic theories—in the form of the dynamic model of the psychological contract (Schalk and Roe, 2007)—and dynamic research methods—in the form of daily diary research and experience sampling research (Fisher and To, 2012)—are available by now, the statistical tools to model the two-stage process are still lacking. The aim of the present paper is to fill this gap by introducing two statistical models that closely mimic the theoretical process underlying the elicitation of violation feelings: the Hurdle model and the Zero-Inflated Regression model. In both models, the two-stage process is represented by two model components: a binary distribution that models whether or not an event has violated one's acceptance limits, and a count distribution that models how severe the negative impact of the violation is. Moreover, covariates can be included for both parts of the model separately, which might yield insight into their unique and shared antecedents. By proposing novel statistical models for the analysis of violation data, the present paper offers a methodological-substantive synergy (Marsh and Hau, 2007), showing how sophisticated methodology can be used to examine an important substantive issue—the elicitation of violation feelings in everyday life.

In what follows, we first discuss the event-based conceptualization of psychological contract violation. Next, we introduce two models that closely align with this event-based conceptualization: the Hurdle regression model and the Zero-Inflated regression model. Apart from a theoretical introduction to these models, we show how these models can be tested using Mplus. Finally, we conclude by comparing our approach to the dominant approaches in the field, discussing how they differ from one another and under which conditions one or the other approach should be used.

## An event-based conceptualization of violation feelings

Although the psychological contract itself—referring to perceived mutual obligations of the employee and employer (Rousseau, 1995)—pertains to a broad exchange process, violations of the psychological contract are more concrete because they are triggered by *events* that happen in relation to work (Conway and Briner, 2002). In line with such an event-based conceptualization of psychological contract violation, Schalk and Roe (2007), in their dynamic model of the psychological contract, conceptualized the psychological contract as a mental model that serves to interpret such events and that therefore lies at the basis of subsequent employee actions and attitudes. The idea of the dynamic model of the psychological contract is that the psychological contract, by providing a frame of reference that offers cues regarding expected events and how to interpret them, eases the evaluation of events taking place. Thus, despite the fact that the psychological contract is always present, Schalk and Roe (2007) argue that it only receives full attention in response to certain events that happen in one's environment.

Importantly, the dynamic model of the psychological contract maintains that not all events lead to violation feelings. In fact, without any major events happening, the psychological contract is in a state of homeostasis, and it is only when the behavior of the organization or the behavior of the employee changes, that the employee will try to accommodate these changes or events within his/her mental model. Crucial for this accommodation process is the idea of acceptance limits, which describe what is considered acceptable for the individual (Schalk and Roe, 2007). Anything that happens within these acceptance limits is perceived as tolerable variation within the agreed-upon contractual obligations, whereas when the acceptance limits are exceeded, the individual feels that the psychological contract has been violated. Indeed, in case the acceptance limits are exceeded, the psychological contract becomes salient to the employee, urging the employee to engage in interpretative processes that might lead to feelings of violation (Morrison and Robinson, 1997).

Thus, the dynamic model of the psychological contract is not build on the assumption that violation feelings result from a “constant method of accounting” in which people systematically and constantly compare perceived promises to perceived obligations (Schalk and Roe, 2007; p. 171). Instead, such feelings are believed to reflect the fact that an event has passed one's acceptance limits, after which interpretative processes are set into motion by the crossing of the limits. This two-sided dynamic conceptualization of violation feelings strongly resembles the distinction Morrison and Robinson (1997) make between breach and violation. In particular, according to Morrison and Robinson (1997) breach “refers to the cognition that one's organization has failed to meet one or more obligations within one's psychological contract in a manner commensurate with one's contributions,” while violation feelings pertain to the “emotional and affective state that may, under certain conditions, follow from the belief that one's organization has failed to adequately maintain the psychological contract” (p. 230).

Of particular importance is that the sense-making process following the crossing of the acceptance limits can happen subconsciously, which implies that employees might experience violation feelings without being consciously aware of the preceding judgments (Morrison and Robinson, 1997). This has also been acknowledged in emotion research, maintaining that, although emotions require cognitive appraisal, the individual does not need to be aware of the factors on which it rests (Lazarus, 1982). This has important implications for the study of violation feelings, because, when the sense-making processes are not always consciously experienced, one cannot expect people to simply reconstruct them or to reliably report on them.

In the present paper, we offer a way to circumvent this thorny issue by presenting dual regime models, a family of statistical models that allow capturing both processes based on one's violation feelings scores only. In what follows, we first give a short theoretical introduction to dual regime models, after which we show how these models can be tested in Mplus using an illustrative application with fabricated data.

## Dual regime models

Assume that we follow different employees in their day-to-day job and that we repeatedly (e.g., each working day) ask them to report on their violation feelings using the following question: “Indicate to what extent you experienced feelings of disappointment, frustration and distress toward your organization today.” People can respond to this question by answering “0 = not at all,” “1 = to a small extent,” “2 = to some extent,” “3 = to a moderate extent,” “4 = to a great extent,” and “5 = to a very great extent.” Because psychological contract breaches do not happen very frequently (i.e., Bal et al., 2017) found that only in 19% of the weeks, participants reported that their psychological contract was violated), the expected distribution of violation feelings scores is one with clumping at zero and relatively few nonzero scores.

Dual regime models are statistical models specifically developed to model data being characterized by clumping at zero. To account for the excess of zeros, these models assume that the data are generated according to two different stages (Zorn, 1996). In the first stage, which is often referred to as the transition stage, the observation moves from a state in which the event of interest does not occur to a stage in which event occurs at a specific rate. In the context of the dynamic model of the psychological contract (Schalk and Roe, 2007), the transition stage thus refers to the crossing of the acceptance limits. After a “successful” transition stage, the observation moves into the events stage, which is the state in which events may occur. Applied to the psychological contract, this means that, once the individual experiences a crossing of his/her acceptance limits, the observation moves to a stage in which feelings of violation might be experienced.

Several dual regime models have been proposed and discussed in the literature, and they can all be classified according to two core model features: (1) the probability distribution that is assumed for the transition stage, and (2) whether or not the events-stage distribution allows for the occurrence of zeros (Zorn, 1996). Especially the second feature is relevant for research on psychological contract violation, because if a model is chosen in which zeros are not generated during the events-stage, one implicitly assumes that a crossing of the acceptance limits should always result in violation feelings. However, when one tests a model in which zeros are generated during the events stage as well, this assumption is relaxed, which means that the acceptance limits can be crossed without such a crossing leading to violation feelings.

Note that the appropriateness of one or the other model is both a theoretical and an empirical issue. Regarding the former, most theoretical accounts indicate that a crossing of the acceptance limits does not necessarily lead to the experiencing of violation feelings. For example, the dynamic model of the psychological contract (Schalk and Roe, 2007) theorizes that a crossing of the acceptance limits causes the psychological contract to become salient to the employee, urging him/her to engage in interpretative processes which might or might not lead to feelings of violation. Similarly, Morrison and Robinson (1997) argue that “… *it is reasonable to assume that employees can perceive that their organization has failed to fulfill an obligation without experiencing the strong affective response associated with the term violation*” (p. 230). Thus, because these theoretical accounts argue that feelings of violation do not necessarily follow from a crossing of the acceptance limits, they would favor a model in which zero violation scores are not only generated in the transition stage, but also in the events stage. At the same time, a model generating zeros in the events stage will not always provide a better fit to the data. For example, imagine that one would conduct a study in an organization that espouses values such as integrity and concern for employees. In such an organization, employees will typically react with more intense violation feelings when a promise is unmet than in an organization that is known to treat employees poorly (Morrison and Robinson, 1997). Hence, each and every crossing of the acceptance limits might result in a non-zero violation score, making a model that does not generate values in the events stage a better fit for the data.

In what follows, we will discuss two dual regime models that differ regarding the allowance of zeros in the events-stage distribution: the Hurdle Poisson Regression Model (no zeros are modeled in the events-stage) and the Zero-Inflated Poisson Model (allowing for the occurrence of zeros in the events stage)^1^. Importantly, both models have a multilevel extension, which is important when studying violation feelings in a dynamic way, using repeated measures data.

### The hurdle regression model

The idea of the Hurdle model is that in the transition stage a “hurdle” needs to be crossed before one moves on to the events stage, which in the context of the dynamic model of the psychological contract maps directly on the crossing of the acceptance limits (Schalk and Roe, 2007). Applied to the violation measure described above, this means that in the transition stage of the Hurdle model, one models whether the rating for feelings of disappointment, frustration and distress toward the organization is zero (i.e., the hurdle is not crossed) or non-zero (i.e., the hurdle is crossed). To this end, the transition stage of the Hurdle model uses a binary logit model in which all counts greater than zero are given value one. This binary logit model can be written as follows:

(1) $$P\left(Violation_{ij}=0\right)=\phi_{ij},\;y=0$$

(2) $$\log\left(\frac{\phi_{ij}}{1-\phi_{ij}}\right)=\gamma_{00}+\gamma_{10}X_{ij}+\ldots +u_{0j}$$

In this model, ϕ_*ij*_ represents the probability of a zero for person *i* on occasion *j*—or the probability of remaining in the zero (no violation feelings) state—, *X*_*ij*_ represents a predictor variable, γ_00_ represents the intercept and *u*_0*j*_ the random effect.

The second part of the Hurdle model pertains to the events stage and describes what happens once the hurdle is taken. At this stage, no more zeros are generated in the Hurdle model. Applied to psychological contract research, this implies that the Hurdle model assumes that, once a person experiences a crossing of the acceptance limits of the psychological contract, the individual will per definition experience violation feelings. Stated differently, in a Hurdle model, violation scores of zero do only result from not crossing the acceptance limits of the psychological contract. Therefore, it is said that, in the Hurdle model, all zeros originate from a “structural” source (Hu et al., 2011).

Because once in the events stage, the individual always experiences violation feelings, the events stage is modeled by a truncated-at-zero count model. Such truncated-at-zero count models are typically used to model count data for processes for which zero is not a possible value. Truncated-at-zero count models can take many forms, with examples being the truncated-at-zero Poisson distribution (Mullahy, 1986) or the truncated-at-zero Negative Binomial distribution (Grogger and Carson, 1991). In the present paper, for reasons of simplicity, we will describe the truncated-at-zero Poisson distribution, although this can easily be extended to a truncated-at-zero Negative Binomial distribution. The formula for the events stage of the multilevel Poisson Hurdle model can be written as follows:

(3) $$P\left(Violation_{ij}=y\right)=(\frac{1-\phi_{ij}}{1-e^{-\lambda_{ij}}})(\frac{e^{-\lambda_{ij}}\lambda_{ij}^{y}}{y!}),\;y>0$$

(4) $$\log\left(\lambda_{ij}^{y}\right)=\beta_{00}+\beta_{10}Z_{ij}+\ldots +\nu_{0j}$$

In formula 3, ϕ_*ij*_ again represents the probability of a zero for person *i* on occasion *j*, while in formula 4, *Z*_*ij*_ represents a covariate, and λ_*ij*_ represents the truncated Poisson mean for counts greater than zero. Finally, ν_0*j*_ captures the random effect.

Relevant to research on psychological contracts is that the Hurdle model allows for the inclusion of common and unique predictors in both stages of the model. That is, one can include predictors that predict violation of the acceptance limits and predictors that predict violation intensity, with the possibility that any of these predictors can be shared and/or unique. Moreover, in the multilevel Hurdle model, the random effect of the binary part and the random effect of the count part can be allowed to correlate. This might make sense from a theoretical point of view, as the presence of a psychological contract violation at one point in time might be related to the intensity of one's violation feelings at that and other points in time. This phenomenon might for example happen when there are (unmeasured and thus unmodeled) person-related factors that influence both the threshold to perceive a violation and the severity of these violation feelings once violation is experienced. One such a person-related factor might be one's level of Neuroticism, because research on this personality traits shows that people scoring high on Neuroticism both experience more negative situations (i.e., more breaches) and also react more strongly to these negative situations (i.e., stronger violation feelings) (Hampson, 2012). Finally, in the multilevel Hurdle model one can also account for potential time-dependencies in the repeated measures data, which is usually done through autoregressive (AR) models (Sutradhar, 2003).

In summary, the Hurdle model is specifically developed to model data generated in two different stages. In the context of psychological contract research, the Hurdle model allows distinguishing between the occurrence of violation feelings and the intensity of the feelings of violation. Moreover, predictors can be included for both violation of the acceptance limits and violation intensity, without requiring that these predictors are the same in both parts of the model. An important characteristic of the Hurdle model is that it assumes that all zeros are generated by failure to cross the hurdle, which means that it assumes that all zero violation feelings scores result from instances where the acceptance limits of the psychological contract were not crossed.

### The zero-inflated regression model

Being a dual regime model, the Zero-Inflated regression model assumes that the data are generated by a two-stage process. However, unlike the Hurdle model, this model assumes that the zero responses are generated by two sources, rather than one. That is, in the Zero-Inflated regression model zeros can arise both in the first stage (i.e., the transition stage) as well as in the second stage (i.e., the events stage). In other words, Zero-Inflated models assume that the zero scores originate from both a “structural” source and a “sampling” source (Hu et al., 2011). Applied to psychological contract research, this means that Zero-Inflated models assume that the absence of feelings of violation (i.e., a violation feelings score of zero) can be due to two reasons. The first (structural) reason is that the acceptance limits were not crossed. The second (sampling) reason is that the acceptance limits were crossed, but that this crossing did not elicit feelings of violation. Thus, according to the Zero-Inflated regression model, one does not need to experience violation feelings when one's acceptance limits of the psychological contract are crossed.

To accommodate the idea that zero violation scores are generated by two different mechanisms or sources, the events stage of the Zero-Inflated models is no longer modeled using a truncated-at-zero count model but using a regular count model. The consequence thereof is that in the event stage, zeros can occur because these zeros are part of the usual count distribution. Very similar to the Hurdle model, Zero-Inflated models can assume different count distributions, such as the Poisson distribution (Lambert, 1992) or a Negative Binomial distribution (Greene, 1994). In what follows, we will discuss the Zero-Inflated Poisson (ZIP) model because of its simplicity and to maximize comparability with our discussion of the Hurdle Poisson model.

Because in the ZIP model, zero scores do not only result from failing to pass the transition stage (i.e., not crossing the acceptance limits of the psychological contract), but also from zeros that are generated during the events stage (i.e., not experiencing violation feelings once the acceptance limits of the psychological contract are crossed), the probability of a zero score is given by the following equation:

(5) $$P\left(Violation_{ij}=0\right)=\phi_{ij}+\left(1-\phi_{ij}\right)e^{-\lambda_{ij}},\;y=0$$

In equation 5, ϕ_*ij*_ represents the probability of a zero for person *i* on occasion *j* during the transition stage, which is the probability of staying in the zero state, while (1 − ϕ_*ij*_) represents the probability of moving to the events stage, or the probability of exceeding the acceptance limits. λ_*ij*_, in turn, governs the intensity of the violation feelings when the acceptance limits are exceeded. Because the ZIP model mixes a binary logit model with a Poisson model, the ZIP distribution can be regarded as a mixture of a Poisson distribution and a degenerate component that places all its mass at zero (Lee et al., 2006)^2^.

The formula for the events stage of the multilevel ZIP can be written as follows:

(6) $$P\left(Violation_{ij}=y\right)=\left(1-\phi_{ij}\right)\frac{\lambda_{ij}^{y}e^{-\lambda_{ij}}}{y!},\;y>0$$

As with the Hurdle model, predictors can be added to both the transition equation (*X*_*ij*_*)* and to the events equation (*Z*_*ij*_*)*, which can be seen below. Note that these predictors can be the same in both parts of the model, but that this is not required. Moreover, as in the multilevel Hurdle model, the random effect of the binary part and the random effect of the count part can be allowed to correlate, and one can account for time-dependencies in the repeated measures data through for example autoregressive (AR) models (Sutradhar, 2003).

(7) $$\log\left(\frac{\phi_{ij}}{1-\phi_{ij}}\right)=\gamma_{00}+\gamma_{10}X_{ij}+\ldots +u_{0j}$$

(8) $$\log\left(\lambda_{ij}^{y}\right)=\beta_{00}+\beta_{10}Z_{ij}+\ldots +\nu_{0j}$$

In summary, the Zero-Inflated Regression model is a dual regime model specifically developed to model data that are generated in two different stages, which, in the context of psychological contract research, allows distinguishing between the occurrence of crossings of the acceptance limits and the feelings of violation that might follow from it. Moreover, predictors can be included for both the crossing of the acceptance limits part and the violation feelings part, without requiring that these predictors are identical in both parts of the model. Unlike the Hurdle model, the Zero-Inflated Regression model assumes that zeros are generated in both the transition stage and the events stage, which means that it assumes that a zero violation feelings scores results either from instances where the individual did not experience a violation of his/her acceptance limits, or from instances where the individual did experience a such a violation but did not experience violation feelings.

## How to test the hurdle model and the zero-inflated regression model: an illustrative application

In what follows, we will demonstrate how the Hurdle model and the Zero-Inflated Regression model can be tested using Mplus version 7.31 (Muthén, 1998-2017). To this end, we make use of fabricated data. These fabricated data have a nested structure with 46 individuals having weekly violation feelings ratings (on a 0–7 scale) for the time course of 4 weeks (*N* = 184 repeated measurements). Sixty four percent of the data have a zero value. Moreover, per individual, the data also contain a trait Neuroticism score. In what follows, we will demonstrate how to test a Multilevel Hurdle Poisson model and a Multilevel Zero-Inflated Poisson model predicting (1) within-person variation in breach and violation feelings using time (in weeks) as a predictor, and (2) between-person variation in breach and violation feelings using Neuroticism as a predictor.

### Testing a multilevel poisson hurdle model using mplus

Mplus does not have an option to directly test the Poisson Hurdle Regression model. However, it can test a Negative Binomial Hurdle Regression model, which is a Poisson model that is extended with a dispersion parameter. This dispersion parameter allows capturing overdispersion in the Poisson model, which means that it allows the variance of the model to be greater than the mean. The Poisson model can thus be approximated by fixing the dispersion parameter of the Negative Binomial Hurdle Regression model to a very small value.

**Figure 1:**
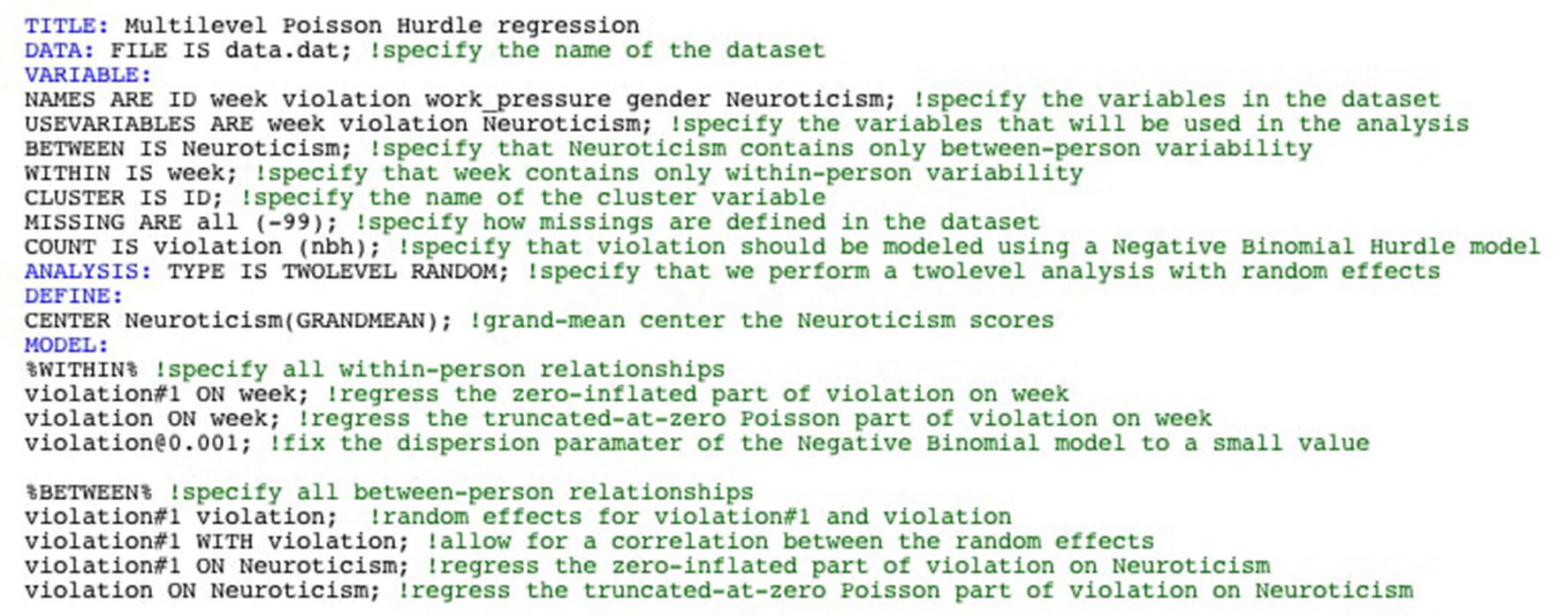
Mplus output for a Multilevel Poisson Hurdle model.

To instruct Mplus to test a Negative Binomial Hurdle Regression model, one needs to specify “COUNT IS violation (nbh);” in the VARIABLE section of the Mplus syntax. Next, one needs to specify that the dispersion parameter of the Negative Binomial Hurdle Regression model should be fixed to a small value by typing “violation@0.001;” under the %WITHIN% header in the MODEL section. This line instructs Mplus to test a Negative Binomial Hurdle Regression model with a very small dispersion parameter, thereby approximating the Poisson Hurdle Regression model. Further, we test the Multilevel Poisson Hurdle model at the within-person level and at the between-person level by in the MODEL section specifying that the zero-inflated part (referred to as violation#1), as well as the count part (referred to as violation) are predicted by week at the within-person level and by Neuroticism at the between-person level. Because of the nested nature of our data, we allow for random effects for both the zero-inflated part and the count part in the model, which is done by typing “violation#1 violation;” at the between-level. Finally, these random effects can be allowed to correlate, which can be done why specifying “violation#1 WITH violation;”. The full Mplus code for testing a Multilevel Poisson Hurdle model can be seen in Figure 1.

### Mplus output for the multilevel poisson hurdle model

**Figure 2:**
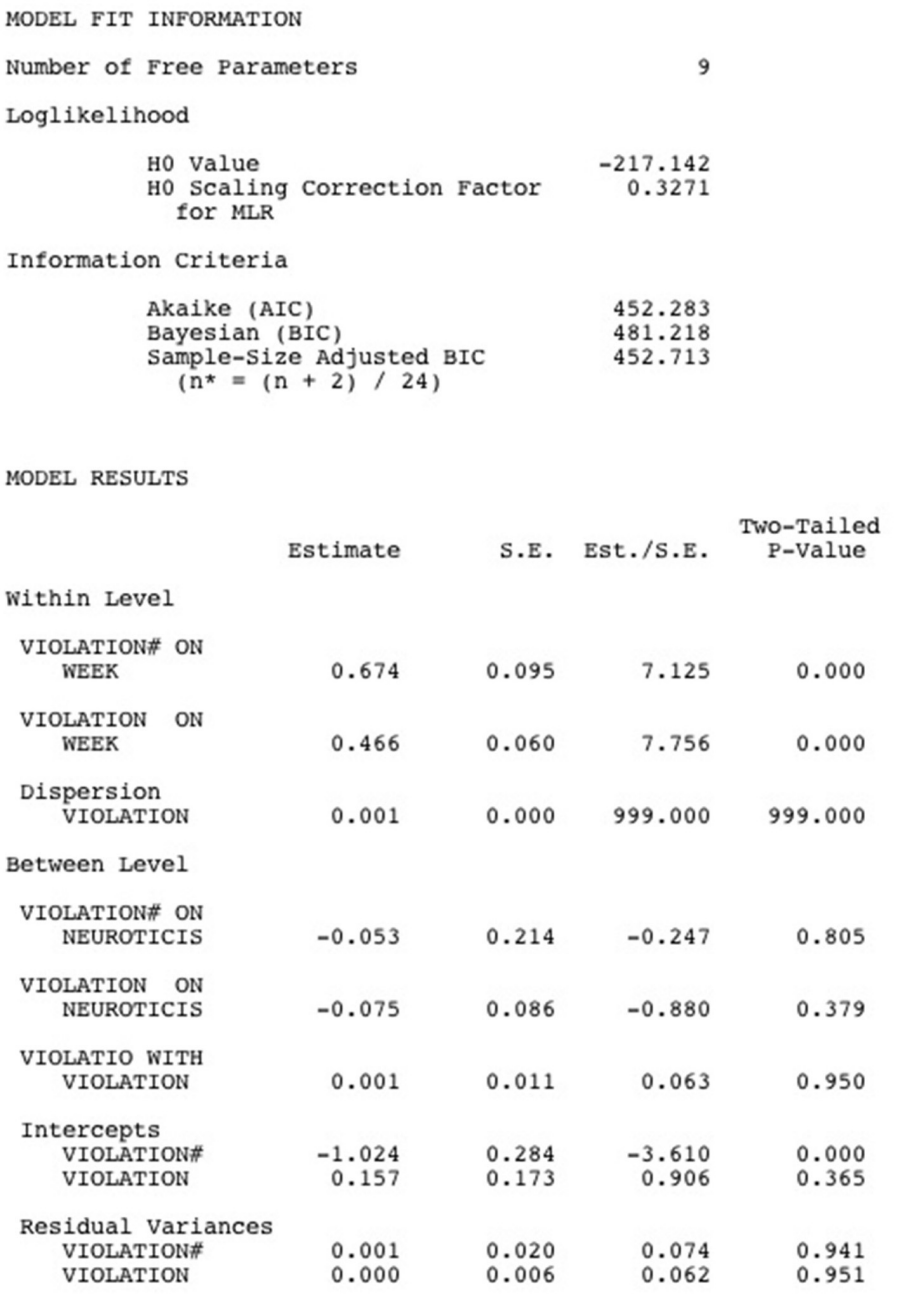
Mplus output for a Multilevel Poisson Hurdle model.

Below, we show the Mplus output for the Multilevel Poisson Hurdle Model (see Figure 2). Under “model fit information,” the loglikelihood of the model and a number of information criteria are shown. All these indices tell how far off the model is from the observed data, which means that lower fit indices are indicative of a better fitting model. However, on their own, the fit indices have little meaning. Only when compared across models, the information criteria can help in model selection. Important to note it that the likelihood value is only appropriate for comparing nested models, while the AIC, BIC and the sample-size adjusted BIC can be used to compare both nested and non-nested models. For all indices, smaller values indicate a better fitting model.

Below the model fit information, the model results are printed. The results at the within-person level show that the chances of *not* crossing the hurdle (i.e., the chance of remaining in the zero violation category) increase as weeks go by (*est*. = 0.674, *p* < 0.001). Moreover, whenever the hurdle is crossed and one experiences violation feelings, these feelings tend to be more intense in later weeks (*est*. = 0.466, *p* < 0.001). The results at the between-person level show that between-person differences in the chance of crossing the hurdle are unrelated to between-person differences in Neuroticism (*est*. = −0.053, *p* = 0.805) and that Neuroticism does not predict between-person differences in violation feelings whenever one experiences breach (*est*. = −0.075, *p* = 0.379). Finally, the random effects for the binary part (*est*. = 0.001, *p* = 0.941) and for the truncated-at-zero count part (*est*. = 0.000, *p* = 0.951) are not statistically significant and they are not correlated to each other (*est*. = 0.001, *p* = 0.950). This implies that there are little between-person differences in the chances of crossing the hurdle and in the intensity of the violation feelings once the hurdle is crossed.

### Testing a multilevel zero-inflated poisson model using mplus

**Figure 3:**
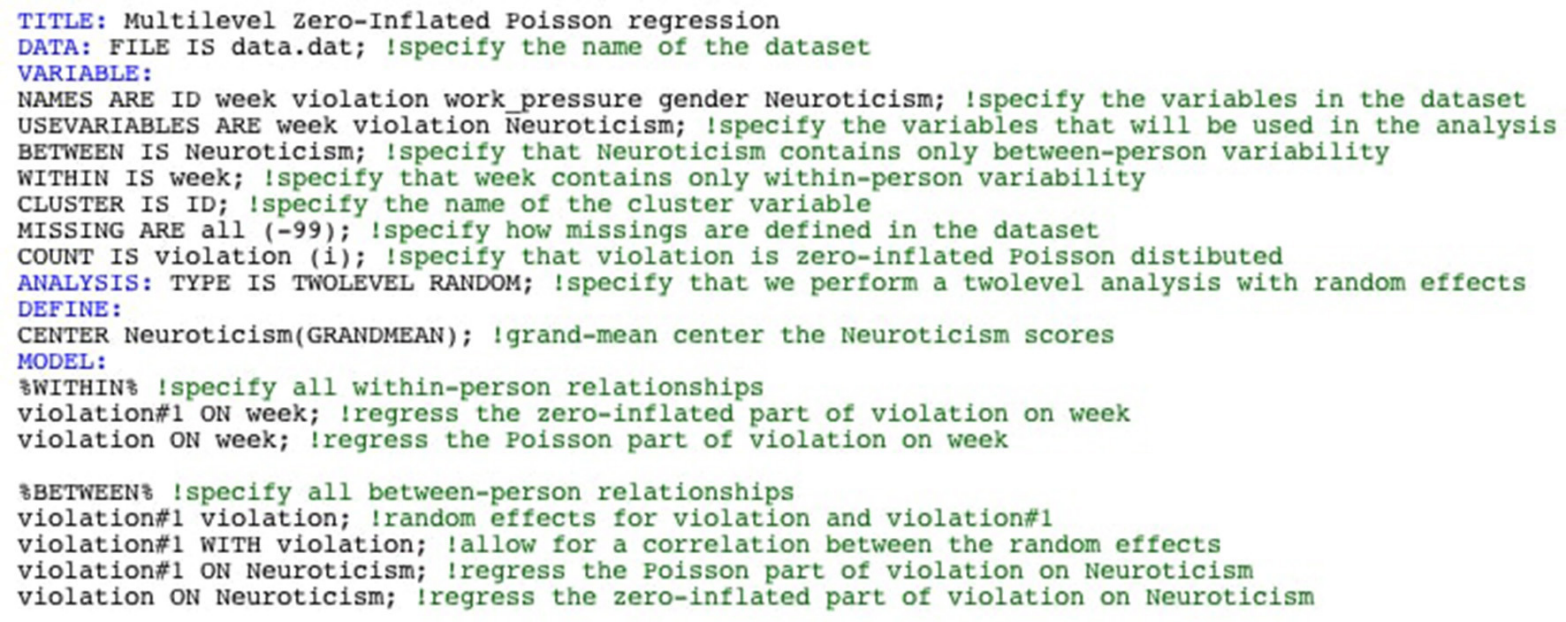
Mplus code for testing a Multilevel Zero-Inflated Poisson model

Telling Mplus to test a Zero-Inflated Poisson Regression model can be done by specifying that violation is a zero-inflated Poisson variable using “COUNT IS violation (i);” in the VARIABLE section of the syntax (note that “COUNT IS violation (nb);” can be used to test a Zero-Inflated Negative Binomial Regression model). Next, one can test the ZIP model at the within-person level and at the between-person level by in the MODEL section specifying that the zero-inflated part (referred to as violation#1), as well as the count part (referred to as violation) are predicted by week at the within-person level and by Neuroticism at the between-person level. Because of the nested nature of our data, we allow for random effects for both the zero-inflated part and the count part in the model, which is done by putting violation#1 and violation at the between-level. Finally, these random effects can be allowed to correlate, which can be done why specifying “violation#1 WITH violation”. The Mplus code for testing a Multilevel Zero-inflated Poisson model can be seen in Figure 3.

### Mplus output for the multilevel zero-inflated poisson regression model

**Figure 4:**
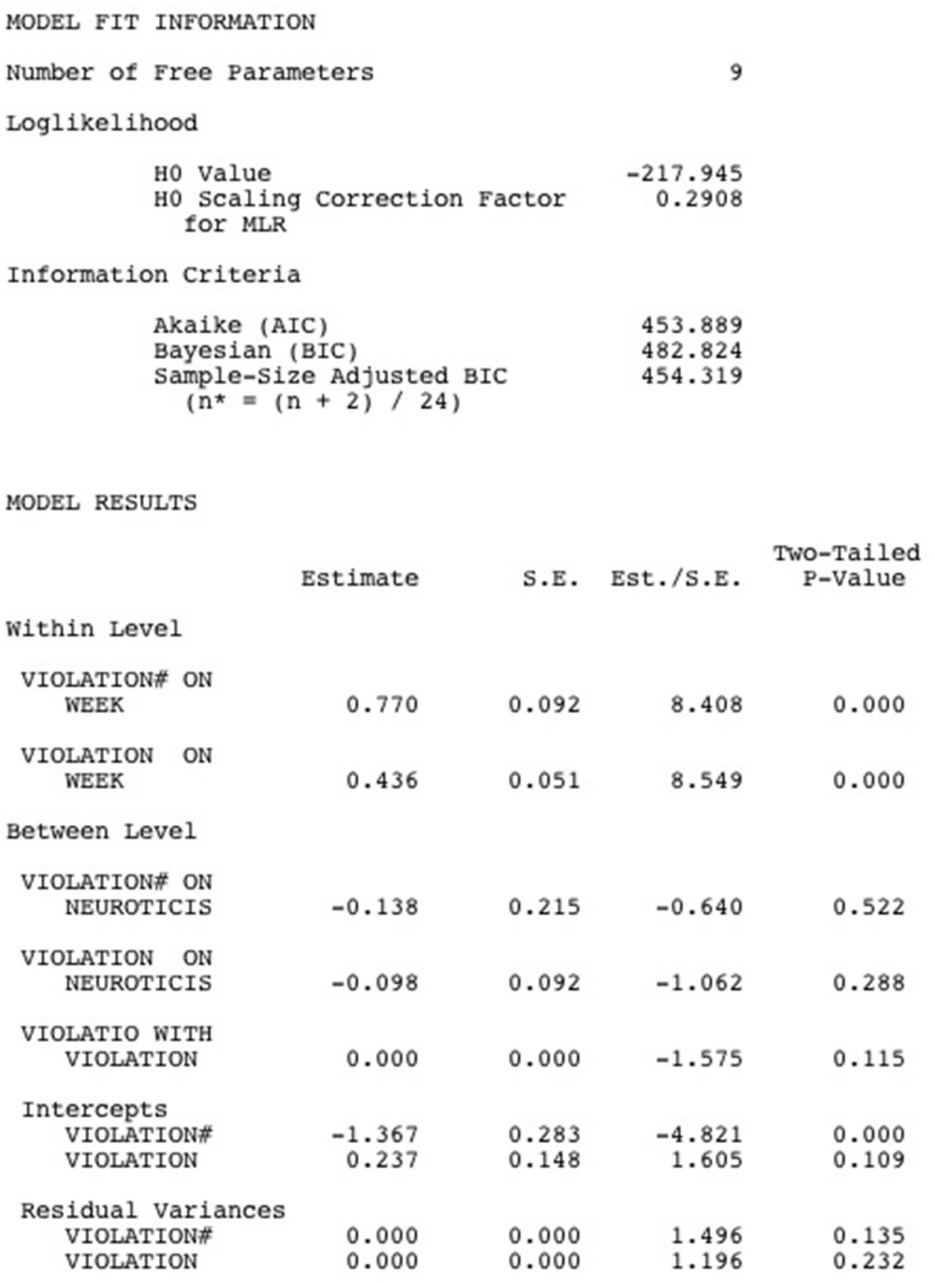
Mplus output for a Multilevel Zero-Inflated Poisson model.

Below, the Mplus output for the Multilevel Zero-Inflated Poisson Regression Model is shown (see Figure 4). Similar to the Hurdle model, under “model fit information,” the loglikelihood of the model and a number of information criteria are printed. As we argued above, the AIC, BIC and sample-size adjusted BIC can be used to compare non-nested models, which means that we can use them to compare the fit of the Multilevel Poisson Hurdle model with that of the Multilevel Zero-Inflated Poisson model. In our example, all information criteria slightly favor the Multilevel Zero-Inflated Poisson model, which means that the competing models test in this case favors a model in which the acceptance limits of the psychological contract can be crossed without this leading to violation feelings.

Next, the model results are printed. The results at the within-person level show that the chances of remaining in the zero state (i.e., the chances of not experiencing breach) increase as weeks go by (*est*. = 0.770, *p* < 0.001). Moreover, violation feelings are more intense in later weeks (*est*. = 0.436, *p* < 0.001). The results at the between-person level show that between-person differences in the chance of remaining in the zero state (i.e., between-person differences in the susceptibility for breach) are unrelated to between-person differences in Neuroticism (*est*. = −0.138, *p* = 0.522) and that Neuroticism also does not predict between-person differences in violation feelings when the acceptance limits are crossed (*est*. = −0.098, *p* = 0.288). Finally, the random effects for the zero-inflated part of the model (*est*. = 0.000, *p* = 0.135) and for the count part (*est*. = 0.000, *p* = 0.232) are not statistically significant and are not related (*est*. = 0.000, *p* = 0.115), implying that there are no significant between-person differences in the susceptibility to breach and in the intensity of violation feelings once breach is experienced.

## Discussion

In the present paper, we argue that dual regime models in general, and Hurdle and Zero-Inflated models in particular, deserve to be added to the toolkit of the psychological contract researcher because these models closely mimic the theoretical processes underlying the elicitation of violation feelings via two model components: a binary distribution that models whether an event in one's work environment leads to a crossing of the acceptance limits of the psychological contract, and a count distribution that models how severe the negative impact of this crossing is. Moreover, covariates can be included for both model parts separately, which might yield insight in their unique and shared antecedents. Hence, the adoption of these models by psychological contract researchers might further our understanding of the factors triggering violation feelings in people's day-to-day working lives.

The treatment of violation feelings by these models strongly draws on a dynamic, event-based conceptualization of the psychological contract, according to which violation feelings follow from discrete events that exceed the acceptance limits of one's psychological contract (Schalk and Roe, 2007). This conceptualization of the psychological contract is at odds with the conceptualization that is adopted by a significant portion of the psychological contract literature up until today. Indeed, the alternative to the event-based conceptualization is one in which it is assumed that violation feelings result from a “constant method of accounting” in which people systematically compare perceived promises to perceived obligations. That some studies hypothesize such a systematic, calculator-like comparison process to operate can be seen the fact that they ask employees to report on their perceived promises and deliveries for a long list of attributes after which polynomial regression analysis is applied to test how the unique interplay of perceived promises and deliveries relates to work outcomes (e.g., Lambert, 2011). Using the same principle, other studies ask employees for each of a range of attributes to indicate whether the organization delivered to them what was promised, after which the scores are aggregated across attributes to create an index (e.g., Turnley and Feldman, 1999). Whereas we focus on discrete crossings of the acceptance limits and momentary feelings of violation, such general measures tap into general, decontextualized violation feelings.

Does this mean that one or the other approach is better than the other? To answer this question it is essential to understand that these different approaches in fact address different questions. Asking people at one point in time to consciously reflect on what was promised to them and what they receive is probably a good way to capture stable, decontextualized inter-individual differences in psychological contract fulfillment and breach. However, because of the one-shot, conscious reflection on promises and deliveries, these studies do probably not reflect the dynamic processes governing psychological breach and violation feelings in people's day-to-day life. The strength of our events-based approach is that it mimics this everyday treatment of violation. At the same time, its weakness is that it fails to tap into the processes leading to inter-individual differences in violation (because it does not measure which obligations are (un)fulfilled). Thus, whether the one or the other approach should be used strongly depends on the questions that are being asked. If one wants to learn about how the different components of the psychological contract contribute to inter-individual differences in violation feelings, the traditional way of conceptualizing and measuring violation is probably appropriate. If, in turn, one is interested in capturing how the psychological contract dynamically operates in people's day-to-day working lives, and if one is interested in studying factors that affect the likelihood to experience violation feelings, an event-based approach is better suited.

It is important to stress that our event-based approach, even though it models dynamic repeated measures data, predicts the occurrence and intensity of violation feelings at one point in time. Another way to look at psychological contact dynamics it to study patterns of violation feelings over time. Recent research by de Jong et al. (2017) in this area shows that, whereas breaches of one's psychological contact have an immediate impact on job-related attitudes, sequentially breached obligations trigger a continuous decline in job satisfaction and citizenship behavior intentions. Moreover, de Jong et al. (2015) showed that cumulative breaches of the psychological contract affect the impact of subsequent breaches on work outcomes. In terms of future research directions, it might be interesting to try to combine both perspectives by for example looking at the effects of cumulative breaches of the psychological contract on the occurrence and the intensity of violation feelings using dual regime models.

When introducing dual regime models, we discussed two of them: the Hurdle Regression model and the Zero-Inflated Regression model. Although we argued that both models can be used to model data generated through a two-stage process, it is important to emphasize that they differ with regard to one crucial aspect. The Hurdle model assumes that all zero observations are structural, which means that this model assumes that, once the acceptance limits of the psychological contract are crossed, the individual per definition experiences violation feelings. The Zero-Inflated model, instead, allows for both structural and sampling zeros, which means that, even when the acceptance limits of the psychological contract are crossed, the crossing might not lead to feelings of violation. Although there are indications in the literature that broken promises do not always lead to violation feelings (Conway and Briner, 2002), it is important to stress that these studies have measured broken promises by asking people (with an open-ended response format) to describe whether the organization has broken any of its promises during the past period (e.g., day or week). The consequence is that, with this procedure, people can report broken promises that were not severe enough to cross the acceptance limits of the psychological contract. This concern is partly supported by the finding that the perceived importance of the promise is one of the crucial factors determining whether violation follows the non-fulfillment of promises (Conway and Briner, 2002). As a result, the question whether violation feelings always follow the crossing of the acceptance limits is not yet settled, and is still in need of further study. One way to investigate this issue might be to systematically compare the fit of the Hurdle Regression model with that of the Zero-Inflated Regression model and see which model fits the data best. Such a model comparison can be done by fitting a series of plausible models to the data (primary candidates are the Hurdle Poisson model and the Zero-Inflated Poisson model), and by systematically comparing their model fit using comparative fit indices such as the AIC, BIC or sample-size adjusted BIC.

In conclusion, when one is interested in studying the ebb and flow of violation feelings in an everyday life context and particularly when the goal is to study predictors of violation feelings, Hurdle Regression model and Zero-Inflated Regression models might be worth looking at. Adopting these methods in research on psychological contracts has the potential to teach us a lot about the features in the situation and the characteristics of the person that trigger dynamic fluctuations in the occurrence and intensity of violation feelings.

## Author contributions

The author confirms being the sole contributor of this work and approved it for publication.

### Conflict of interest statement

The author declares that the research was conducted in the absence of any commercial or financial relationships that could be construed as a potential conflict of interest.

## Footnotes

^1^Note that the Hurdle Poisson Regression Model and the Zero-Inflated Poisson Model are specific cases of the Hurdle Regression Model and the Zero-Inflated Model in the sense that the events stage is modeled using a truncated-at-zero Poisson and a regular Poisson distribution, respectively. For both the Hurdle and Zero-inflated model, other distributions can be used for modeling the events stage, with one example being the negative binomial distribution. However, because of reasons of simplicity, we primarily focus on the Poisson variants in the present paper.

^2^Note that this is different from the Hurdle model. In the Hurdle model all zero scores are modeled using a binary logit model, while all nonzero scores are modeled using a truncated-at-zero Poisson mode. Hence, one can test the Hurdle model by testing both models separately (i.e., a two-stage analysis). This is not possible with the Zero-Inflated Poisson model because only part of the zero scores are explained by the binary logit model, while the other zero scores are accounted for by the Poisson distribution. Therefore, both model equations need to be estimated simultaneously.
